# Hydrolyzed Protein Formula in Very-Low-Birth-Weight Infants: Predictors of Use and 20-Month Neurodevelopmental Outcome

**DOI:** 10.7759/cureus.79828

**Published:** 2025-02-28

**Authors:** Kousiki Patra, Jieun David, Michelle M Greene

**Affiliations:** 1 Pediatrics and Neonatology, Rush University Children's Hospital, Chicago, USA; 2 Pediatrics, Endeavor Health, Chicago, USA; 3 Pediatrics, Rush University Children's Hospital, Chicago, USA

**Keywords:** infant development, infant formulas, infant nutrition, preterm infant, vlbw infants

## Abstract

Objectives: Hydrolyzed protein formulas (HPFs) are used in full-term infants to treat cow’s milk protein allergy but can result in suboptimal weight gain as compared to standard infant formulas. In preterm infants, HPF may be used to promote feeding tolerance after necrotizing enterocolitis. There is limited evidence on HPF use in preterm infants after NICU discharge. The primary objective of this study was to determine predictors of HPF use at four, eight, and 20 months corrected age (CA) post-NICU discharge, and the secondary objective was to evaluate the association between HPF use at four and eight months CA and neurodevelopmental (ND) outcome (cognitive, language, and motor indices on the Bayley Scales of Infant and Toddler Development-III) at 20 months CA in very-low-birth-weight (VLBW; birth weight < 1,500 g) infants.

Methods: This was a retrospective chart review of 419 VLBW infants born in 2008-2012. Infants were categorized by diet at four and eight months into one of three groups: any maternal breast milk (MBM), HPF, and non-HPF. ND outcome was assessed with the Bayley-III. Multiple regression adjusted for the effect of risk factors on formula use and the effect of four- and eight-month diets on 20-month ND.

Results: Forty-three (10.3%), 45 (10.7%), and 350 (83.5%) infants at four months and 22 (5.5%), 41 (10.3%), and 297 (74.4%) infants at eight months were on a diet of MBM, HPF, and non-HPF, respectively. HPF use was predicted by multiple gestation (odds ratio (OR) 3.01 (95% CI 1.57-5.99)) and stage 2-3 necrotizing enterocolitis (OR 2.54 (95% CI 1.09-5.94)) at four and eight months, respectively. In multiple regression, infants on non-HPF at eight months had worse language/receptive language scores than the other two groups.

Conclusions: In our retrospective analysis of VLBW infants, we did not observe any statistically significant difference in 20-month ND outcomes among infants who received HPF as compared to those who received non-HPF or MBM.

## Introduction

Hydrolyzed cow’s milk protein formulas have been used in full-term infants to treat feeding intolerance and are used as a primary nutrition source in infants with a diagnosis of cow’s milk protein allergy [1]. In preterm infants, hydrolyzed protein formula (HPF) may be used to promote feeding tolerance, especially after necrotizing enterocolitis (NEC) [2-9]. HPFs are termed partially or extensively hydrolyzed based on the degree of hydrolysis of proteins (molecular weights of 3.0-10.0 kDa vs. <1.5-3.0 kDa, respectively) [8]. Although they are thought to improve gastrointestinal tolerance by accelerating gastric emptying, reducing intestinal transit, and stimulating enzymatic activity and digestion, there is conflicting data on whether this translates to shorter time to full feedings in preterm infants [2,6].

Postnatal growth is essential to preterm infant outcomes, with poor growth associated with worsened cognitive and educational outcomes [8]. Preterm infants who experience feeding intolerance or interrupted feedings are particularly vulnerable to nutritional deficits and postnatal growth restriction [8]. Studies examining feeding intolerance and time to enteral feeds in preterm infants on the HPF diet report conflicting findings, with various studies reporting shortened time versus no significant difference in time to full enteral feeds [2,6,7]. Recent data has demonstrated no difference in nitrogen balance and plasma amino acid concentrations in comparison to standard preterm infant formula [8]. With regard to growth, observations of decreased weight or lower growth velocity in infants on HPF [8,10] versus no observed changes in childhood growth parameters in comparison to those on standard formula have been reported [11-14].

Given the potential implications of HPF usage in this vulnerable population, we sought to explore gaps in understanding the long-term usage and influences of HPF. Furthermore, we hypothesized that infants on HPF would have worse neurodevelopmental (ND) outcomes as compared to infants on maternal breast milk (MBM). The primary objective of this study was to determine predictors of HPF use at four, eight, and 20 months corrected age (CA) post-NICU discharge, and the secondary objective was to evaluate the association between HPF use at four and eight months CA and ND outcomes (cognitive, language, and motor indices on the Bayley Scales of Infant and Toddler Development-III (BSITD-III)) at 20 months CA in very-low-birth-weight (VLBW; birth weight (BW) < 1,500 g) infants.

## Materials and methods

**Figure 1:**
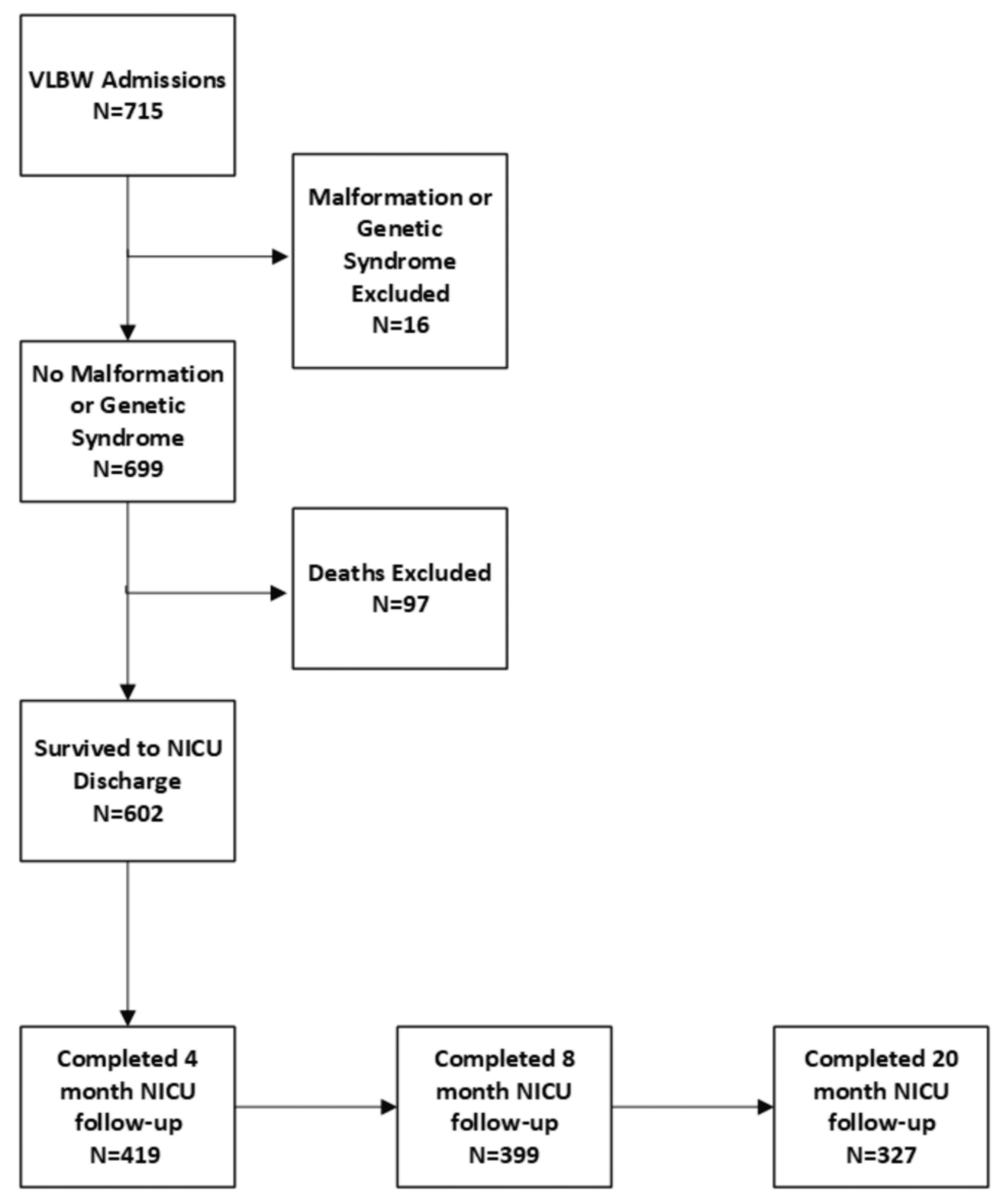
Study Population VLBW: very-low-birth-weight

We conducted a retrospective analysis via manual electronic chart review of all VLBW infants born in 2008-2012 who were admitted to the Rush University Medical Center (RUMC) NICU and had completed at least one visit to the RUMC Neonatal High-Risk Follow-up Clinic after discharge. All data was retrieved from the electronic medical records. Infants were excluded if they had a major congenital malformation or genetic disorder, were transported to another facility prior to NICU discharge, or did not attend the NICU follow-up clinic (see Figure 1). During the study period, it was standard care to evaluate all VLBW infants at four, eight, and 20 months CA in the Neonatal High-Risk Follow-up Clinic, a multidisciplinary clinic that monitors the growth and neurologic and developmental status of infants cared for in the NICU. Data on diet was obtained from verbal parental reports by the provider at each visit and reflected the diet the child was at the time of the visit categorized into one of three groups: any amount of MBM, exclusive diet of HPF (including partially and/or extensively hydrolyzed and free amino acid formulas), and exclusive diet of non-HPF. This study was conducted according to the guidelines laid down in the Declaration of Helsinki, and all procedures involving human subjects were approved by the Institutional Review Board of RUMC (14102106-IRB01). Informed consent was exempt due to minimal risk of harm.

Nutritional practices in the NICU

For all three groups of infants, parenteral nutrition was started at birth and weaned as feedings were advanced and discontinued when enteral feeding volume was at 120 mL/kg/day. Enteral feedings were started as soon as MBM was available by day of life 4-5 after which non-HPF (20 kcal per ounce) was started. During these study years, donor milk was not used in the RUMC NICU. For all study years, non-HPF was changed to 24 kcal per ounce, and MBM feedings were fortified with powdered multinutrient, intact-protein human milk fortifier (Similac Human Milk Fortifier, Abbott Nutrition, Lake County, IL, US) when enteral feedings reached 140 mL/kg/day. The target enteral feeding volume was 160 mL/kg/day unless changed by the provider. Infants were discharged on unfortified MBM or 22 kcal per ounce transitional non-HPF. During their hospitalization, infants were changed to an HPF diet at the discretion of the attending neonatologist for indications such as NEC or feeding intolerance.

Birth, neonatal, and sociodemographic data

Collected birth and neonatal data included the delivery mode, BW, gestational age (GA), infant sex, small for GA (SGA) at birth, multiple gestations, severe brain injury (grade 3-4 intraventricular hemorrhage, periventricular leukomalacia, or hydrocephalus), sepsis (culture-proven blood or cerebrospinal fluid), NEC (stages 2 and 3 according to Bell et al.), bronchopulmonary dysplasia (BPD; oxygen or positive pressure ventilation at 36 weeks CA), treatment with postnatal steroid (yes/no) and treatment (medical or surgical) for patent ductus arteriosus (PDA), and any stage of retinopathy of prematurity (treated or with spontaneous regression) [15,16]. Collected sociodemographic data included maternal race/ethnicity, age, and type of health insurance (public or private).

ND outcome data

Infants were evaluated at 20 months CA by a neonatologist and psychologist. Assessments included a medical history, a neurologic exam according to Amiel-Tison, and the BSITD-III [17,18]. The BSITD-III provides age-adjusted standard scores for cognitive, language, and motor skills (mean 100 ± 15) and subtest scores for cognitive, receptive language, expressive language, fine motor, and gross motor subscales (mean 10 ± 3) based on nationally representative normative data [18]. The primary outcomes of this study included continuous cognitive, language, and motor index scores. During the study years, the BSITD-III were administered by one of two pediatric developmental neuropsychologists with specialized training in the Bayley, with one having been trained directly by the other.

Data analysis

For neonatal, social, and feeding data, measures of central tendency and frequencies were used to provide descriptive data. Chi-squared test and ANOVA analyzed social and neonatal variables by feeding group (any MBM, exclusive HPF, and exclusive non-HPF). Multivariable logistic regression analyses were then performed to examine the associations between social and neonatal variables and HPF use at four and eight months CA. Additionally, independent-sample t-tests and Chi-squared tests analyzed the associations between social, neonatal, and feeding variables and follow-up attendance at the 20-month CA follow-up visit.

For ND outcome data, multivariable linear regression analyses examined the associations between feeding groups and BSITD-III cognitive, language, and motor composite index scores after accounting for relevant attendance variables (entered in step 1, variables with p-values < 0.05 retained in step 2/final model) and social, neonatal, feeding, and ND covariates (step 2/final step; Statistical Package for the Social Sciences (SPSS) version 23.0, IBM Corp., Armonk, NY, US). Finally, Chi-squared tests were employed for exploratory analyses of associations between feeding and ND outcome classified as BSITD-III index scores of <85 and <70. Multivariable logistic regression analyses examined associations between feeding groups and BSITD-III <85 and <70 after accounting for relevant attendance variables (entered in step 1, variables with p-values < 0.05 retained in step 2.final model) and social, neonatal, feeding, and ND covariates (step 2/final).

## Results

Sociodemographic and neonatal data

The details of the patient population are shown in Figure 1. Four hundred and nineteen (69.6%) of 602 infants completed at least one visit to the NICU follow-up clinic (Table 1) while 327 (54.3%) completed follow-up through 20 months CA. Of the 327 infants who completed the 20-month CA visit, 203 (62.1%) received postnatal steroids, 209 (63.9%) had BPD, and 252 (77.1%) received MBM at discharge as compared to 77 (28.0%), 99 (36.0%), and 63 (22.9%) of the 275 infants who did not attend the 20-month visit, respectively. There were significantly more multiples in the HPF group as compared to infants in the MBM or non-HPF group (Table 1). There were significant socioeconomic differences between the three groups in the categories of being young mothers (with the non-HPF group significantly more likely to be born to younger mothers), having public health insurance (with the highest percentage in the non-HPF group and the second highest in the HPF group), and being of Black race (with both the HPF and non-HPF groups with higher percentages as compared to the MBM group) (Table 1). There were no significant differences in neonatal characteristics among the groups other than NEC and/or intestinal perforation, which was present in nine (23.1%) of infants in the HPF group as compared to only four (9.8%) and 30 (8.8%) of the MBM and non-HPF groups, respectively.

**Table 1:** Birth, Sociodemographic, and Neonatal Characteristics of Feeding Groups *Significant p-value MBM: maternal breast milk; HPF: hydrolyzed protein formula; SD: standard deviation; NEC: necrotizing enterocolitis

Variable	Any MBM (n =4 1)	HPF (n = 39)	Non-HPF (n = 339)	p-value
	N (%) or mean ± SD			
Birth weight (g)	989 ± 258	1,049 ± 256	1,033 ± 269	0.54
Birth gestational age (weeks)	28.4 ± 3	28.4 ± 2	28.2 ± 2.6	0.90
Male gender	14 (34.2%)	20 (51.3%)	175 (51.6%)	0.10
Race	-	-	-	0.03*
White	16 (39.0%)	14 (35.9%)	78 (23.0%)	-
Black	12 (29.3%)	19 (48.7%)	171 (50.4%)	-
Hispanic	12 (29.3%)	6 (15.4%)	87 (25.7%)	-
Multiple birth	14 (34.2%)	18 (46.2%)	68 (20.1%)	<0.001*
Small for gestational age	12 (29.3%)	5 (12.8%)	52 (15.3%)	0.06
Antenatal steroids	36 (87.8%)	37 (94.9%)	298 (87.9%)	0.18
Cesarean section	32 (78.1%)	30 (76.9%)	223 (65.8%)	0.13
Maternal age	31.3 ± 4.9	30 ± 6.6	27.5 + 6.7	<0.001*
Public health insurance	16 (39.0%)	21 (53.9%)	248 (73.2%)	<0.001*
Severe brain injury	1 (2.4%)	3 (7.7%)	34 (10.0%)	0.27
Sepsis	2 (4.9%)	6 (15.4%)	41 (12.1%)	0.30
NEC/intestinal perforation	4 (9.8%)	9 (23.1%)	30 (8.9%)	0.021*
Treated patent ductus arteriosus	16 (39.0%)	14 (35.9%)	142 (41.9%)	0.91
Bronchopulmonary dysplasia	14 (34.2%)	17 (43.6%)	136 (40.1%)	0.65
Postnatal steroids	10 (24.4%)	11 (28.2%)	94 (27.7%)	0.91
Retinopathy of prematurity	11 (26.8%)	5 (12.8%)	70 (20.7%)	0.30
Corrected age at discharge (weeks)	39 ± 2.7	40 ± 6	39 ± 3.4	0.16

Feeding data

Data on diet after NICU discharge is presented in Table 2. At four months CA, only 24 (5.7%) were on a diet of exclusive MBM while 19 (4.5%) were receiving MBM supplemented with HPF (n = 4) or non-HPF (n = 15). Two hundred and sixty-two (62.5%) of infants were receiving preterm infant transitional formula, 69 (16.5%) were receiving a term infant cow’s milk formula, and 12 (2.9%) were receiving soy-based formulas (Table 2). Forty-five (10.7%) of infants were receiving HPF, the majority of which were partially or extensively hydrolyzed formulas at the four-month CA visit. Fifty-one percent of infants on HPF at four months CA remained on HPF at eight months CA. However, 29% of infants on HPF at eight months CA were started on it after the four-month CA visit. At 20 months CA, only five (1.5%) of infants seen remained on HPF while the majority of infants, 291 (89.0%), were on a diet of cow’s milk. Multiple regression analyses revealed that multiple birth and history of NEC were significant predictors of HPF use: multiple births were three times more likely to use HPF at four months CA (odds ratio (OR) 3.01 (95% CI 1.57-5.99)) while infants with a history of NEC or intestinal perforation were 2.54 times more likely to continue to use HPF at eight months CA (OR 2.54 (95% CI 1.09-5.94)) (Table 3).

**Table 2:** Diet at 4, 8, and 20 Months Corrected Age

Diet	4 months (N = 419)	8 months (N = 399)	20 months (N = 327)
	N (%)		
Exclusive maternal breast milk	24 (5.7%)	15 (3.8%)	1 (0.31%)
Maternal breast milk + formula	19 (4.5%)	7 (1.8%)	-
Preterm infant cow’s milk transitional formula	262 (62.5%)	178 (44.6%)	-
Term cow’s milk formula	69 (16.5%)	104 (26.1%)	2 (0.61%)
Hydrolyzed protein formula	45 (10.7%)	41 (10.3%)	5 (1.5%)
Partially hydrolyzed	20 (44.4%)	19 (46.3%)	0
Extensively hydrolyzed	20 (44.4%)	16 (39.0%)	2 (40.0%)
Amino acid formulas	5 (11.1%)	6 (14.6%)	3 (60.0%)
Soy infant formula	12 (2.9%)	11 (2.8%)	-
Formula for gastroesophageal reflux	7 (1.7%)	4 (1.0%)	-
Cow’s milk	-	41 (10.3%)	291 (89.0%)
Cow’s milk toddler formula	-	3 (0.75%)	15 (4.6%)
Alternate milk beverage	-	1 (0.25%)	10 (3.1%)

**Table 3:** Predictors of HPF Use at 4 and 8 Months Corrected Age NEC: necrotizing enterocolitis; GA: gestational age; HPF: hydrolyzed protein formula

Variable	4 months	8 months
	Odds ratio (95% CI)	
Multiple birth	3.01 (1.57, 5.99)	1.62 (0.77, 3.39)
NEC/intestinal perforation	2.16 (0.90, 5.15)	2.54 (1.09, 5.94)
Corrected GA at discharge	1.05 (0.96, 1.13)	1.05 (0.98, 1.12)

ND outcome data

**Figure 2:**
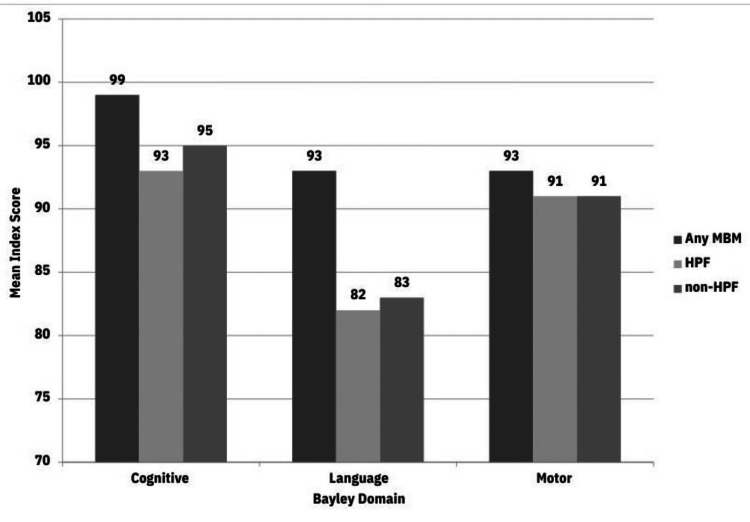
BSITD-III Index Scores at 20 Months Corrected Age BSITD-III cognitive, language, and motor index scores at 20 months corrected age for the any MBM, HPF, and non-HPF groups. BSITD-III: Bayley Scales of Infant and Toddler Development-III; MBM: maternal breast milk; HPF: hydrolyzed protein formula

There were no differences in the 20-month CA BSITD-III index scores between the three groups according to the four-month CA diet. However, infants exclusively on HPF or non-HPF at eight months CA had significantly lower mean language index scores compared to infants on any MBM (p < 0.05, Figure 2) at 20 months CA. In linear regression analyses on eight-month CA diet and 20-month CA BSITD-IIII outcomes, only the non-HPF group demonstrated significantly lower language scores as compared to infants on MBM (unstandardized b = -8.6 (-16.0, -1.1), p < 0.05). Infants on the HPF diet demonstrated lower language scores compared to infants on MBM also (unstandardized b = -7.8 (-16.9, 1.3), p = 0.094), but this did not reach statistical significance. Subgroup analyses of BSITD-III index scores <70 and <85 did not reveal any association between feeding groups at four-month CA and 20-month CA outcomes. Infants who received any MBM at eight months CA were less likely to have language indices < 85 at 20 months CA. In logistic regression analysis, infants with public health insurance were 2.3 times more likely to have a language index more than 1 standard deviation (SD) below normal at 20 months CA (p = 0.009), and infants continuing to receive MBM at eight months CA were 73% less likely to have a language index more than 1 SD below normal at 20 months CA (p = 0.042).

## Discussion

HPFs have been used extensively in preterm infants in the past several decades to help promote feeding tolerance, shorten intestinal transit time, reduce the risk of gastroesophageal reflux (GER) disease, and prevent sensitization to cow’s milk protein and reduce atopy [2-8]. To our knowledge, this is the only study to examine predictors of HPF use among preterm infants after NICU discharge and the first to examine the relationship between HPF and ND outcomes in VLBW infants. In examining predictors of use, multiple birth was the most significant risk factor for HPF use at four months CA while a history of NEC or intestinal perforation predicted HPF use at eight months CA. Although supplementation with non-HPF formula was the standard procedure in the RUMC NICU for hospitalized infants, it remains possible that the relationship found between multiple gestation and HPF usage is related to breast milk unavailability and concern for feeding tolerance. Infants of multiple gestation may be provided the same formula for ease of feeding or for concern for feeding issues if already presenting in one infant. While data on feeding intolerance or GER episodes were not collected in this study, GER symptoms were also frequently reported by families as the indication for changing their infant’s formula from non-HPF after NICU discharge. Corvaglia et al. investigated the role of HPF in reducing GER in preterm infants and reported that in a randomized trial, HPF significantly reduced the number of GER episodes detected by pH monitoring and the reflux index [4]. Further studies are needed to understand the association between GER and feeding intolerance, particularly following NICU discharge.

Within NICUs, refeeding infants recovering from NEC is a frequent indication of the use of HPF in hospitalized infants to improve absorption and feeding tolerance [4,5,7]. A survey study of European surgeons by Zani et al. found that when breast milk is unavailable, infants are fed HPF formulas or amino acid-based formulas following NEC presentation [19]. Similarly, a history of NEC was found to be a predictor of longer-term HPF usage. Given the lack of standardized guidance on change in formula following diagnosis of NEC, these findings suggest that infants may continue for extended periods of time on HPF. Studies have reported a suspected relation between cow’s milk protein allergy and NEC [3,5], which may contribute to the longer-term usage of HPF in infants with a history of NEC.

Regarding ND outcomes, both the HPF and non-HPF groups demonstrated significantly lower mean language index scores at 20 months CA compared to infants on any MBM, and any MBM intake corresponded to a lower likelihood of subnormal language index. The impact of MBM on cognition has been demonstrated in numerous studies, with improvements in verbal intelligence quotient (IQ) demonstrated with increased MBM intake [20-23]. It is important to also note the significant differences in the percentages of younger mothers, public health insurance, and Black race in the non-HPF and HPF groups in comparison to the any MBM group. Socioeconomic inequities yield differential outcomes in child development [24,25], with poorer developmental outcomes noted in the preterm population in the setting of socioeconomic risk factors [26,27]. In our findings, infants with public health insurance were significantly more likely to have a subnormal language index. These multiple factors likely contribute to this large difference observed in BSITD-III testing at 20 months CA in the MBM group.

Although there was a trend toward lower language scores with infants in the HPF group, only non-HPF diet at eight months CA was significantly associated with adverse language outcomes at 20 months CA after regression analyses. There was no association seen between diet at four months CA and ND at 20 months CA. It is possible these differing findings are related to study limitations, including the smaller number of infants in the HPF group or the inability to collect the duration of a specific diet. Nevertheless, these findings suggest that there are no meaningful longitudinal consequences on ND outcomes related to HPF, particularly when compared to the non-HPF diet. Two prior studies have examined ND outcomes related to HPF usage in less vulnerable populations. In Mennella et al.’s randomized controlled trial (RCT) of 79 healthy term infants randomized to cow milk formula versus partial or exclusive HPF diet, they found both HPF-fed groups had higher gross motor and visual reception scores at 5.5-8.5 months while higher receptive language scores were seen for the cow milk formula-fed infants versus the infants fed solely HPF [28]. However, the only difference seen at the end of their 8.5-month observation period was higher visual reception scores seen in the exclusive HPF group [28]. Somaraki et al. followed the ND of 6,979 infants at 33 weeks GA or greater who were fed non-HPF and partially or extensively HPF [29]. Though they observed decreased gross motor skills at 1-year follow-up in infants fed HPF, this association was not seen at 1- and 3.5-year follow-up [29]. They also found that the degree of protein hydrolysis was not associated with ND scores up to 3.5 years [29]. Similarly, our findings do not suggest an unfavorable association between HPF usage and ND outcomes in early childhood. Our study adds to the growing body of literature reflecting the safety of HPF on ND outcomes, particularly in a high-risk population.

The strengths of our study are a relatively large cohort of VLBW infants for whom we had a large amount of sociodemographic, neonatal, and dietary information, allowing us to adjust for differences in characteristics between the feeding groups. Despite our attrition rates for follow-up, we had detailed ND data through standardized assessment with the BSITD-III, and to date, there are no published studies on ND outcomes in VLBW infants exposed to HPF. Also, we adjusted for differences between those who attended follow-up vs. those who did not. However, our study has several limitations, namely, that it is a single-center retrospective, older cohort and not a RCT of HPF and non-HPF diet. Initiation of HPF was at the attending neonatologist's discretion and not based on a uniform practice guideline. Although there were relatively few changes made in nutritional practices during the period, we cannot control for all the practice changes that may have occurred during this time. Additionally, there were significant differences between the three groups of patients. Infants in the HPF group were more likely to be a multiple and have a history of NEC while infants in the MBM group were born to older mothers who were more likely to have private health insurance. These findings could have significant implications on the home environment after NICU discharge, including access to reading, play-based activities, and educational resources and therapies known to impact ND outcomes. However, there were no differences in rates of other neonatal morbidities between the groups, and results were adjusted for differences in characteristics among the groups. Although we were able to collect feeding data from the NICU follow-up clinic visits, all data was by parent report and was reflective only of the diet at the time of the visit and not a dietary log of the previous months. As such, we did not have data on the duration/volume of each of the feedings. Similarly, reporting on growth parameters related to feedings was outside the scope of this study. Furthermore, we had significant attrition from four months to 20 months CA. Infants who attended the 20-month CA visit were more likely to have BPD, be on a diet of MBM at discharge, and have older mothers. Although these differences were adjusted for, it is possible that this may have impacted our ND outcome results. Finally, as this was a single-center study, it is unclear how generalizable these findings are to centers/populations with higher utilization of HPF and non-HPF. Despite these limitations, this study provides novel information on predictors of long-term HPF use in preterm infants and the relationship of HPF use with ND outcomes in the VLBW population. These findings may help inform clinicians and researchers regarding the nutritional management of preterm infants after NICU discharge.

## Conclusions

In this retrospective, single-center study, we found that multiple gestation was the most significant risk factor for the use of HPF at four months CA, while a history of NEC was associated with a persistence of HPF use at eight months CA in VLBW infants after NICU discharge. Although both the non-HPF diet and the HPF diet were associated with worse language outcomes at 20 months CA compared to those on the MBM diet, only the non-HPF diet was associated with abnormal ND outcomes at 20 months CA after linear regression analyses. We acknowledge that our sample size, study attrition, and reliance on parental reports for diet are limitations of this study. Future prospective studies with detailed NICU and postdischarge dietary histories are necessary to delineate the impact of HPF feedings on ND outcomes, and future studies should account for environmental factors that can affect ND domains such as language.
